# Study of the Ghrelin/LEAP-2 Ratio in Humans and Rats during Different Phases of Pregnancy

**DOI:** 10.3390/ijms23179514

**Published:** 2022-08-23

**Authors:** Maria Fernanda Garcés, Julieth Daniela Buell-Acosta, Edith Ángel-Müller, Arturo José Parada-Baños, Jaidy Acosta-Alvarez, Harold Felipe Saavedra-López, Roberto Franco-Vega, Luis Miguel Maldonado-Acosta, Franklin Escobar-Cordoba, Keydy Vásquez-Romero, Ezequiel Lacunza, Sofía Alexandra Caminos-Cepeda, Rubén Nogueiras, Carlos Diéguez, Ariel Iván Ruiz-Parra, Jorge Eduardo Caminos

**Affiliations:** 1 Department of Physiology, School of Medicine, Universidad Nacional de Colombia, Bogotá 11001, Colombia; 2 Department of Gynecology and Obstetrics, School of Medicine, Universidad Nacional de Colombia, Bogotá 11001, Colombia; 3 Department of Morphology, School of Medicine, Universidad Nacional de Colombia, Bogotá 11001, Colombia; 4 Endocrinology Unit, Department of Internal Medicine, School of Medicine, Universidad Nacional de Colombia, Bogotá 11001, Colombia; 5 Psychiatry Department, School of Medicine, Universidad Nacional de Colombia, Bogotá 11001, Colombia; 6 Fundación Sueño Vigilia Colombiana, Bogotá 111211, Colombia; 7 Centro de Biomodelos CeBio, Departamento de Ciencias Biológicas, Universidad de los Andes, Bogotá 111711, Colombia; 8 Centro de Investigaciones Inmunológicas Básicas y Aplicadas (CINIBA), Facultad de Ciencias Médicas, Universidad Nacional de La Plata, La Plata 1900, Argentina; 9 School of Medicine, Universidad Pompeu Fabra, 08002 Barcelona, Spain; 10 CIBER Fisiopatología de la Obesidad y Nutrición, Instituto de Salud Carlos III, 28029 Madrid, Spain; 11 Department of Physiology (CIMUS), School of Medicine, Instituto de Investigaciones Sanitarias (IDIS), Universidad de Santiago de Compostela, 15782 Santiago de Compostela, Spain

**Keywords:** Ghrelin/LEAP-2 ratio, pregnancy, endogenous GHSR antagonist

## Abstract

The Liver-Expressed Antimicrobial Peptide 2 (LEAP-2) has emerged as an endogenous GHS-R antagonist and blunts the orexigenic action of ghrelin. This study aimed to determine the Ghrelin/LEAP-2 ratio in humans and rats during pregnancy. In humans, we conducted a nested case-control study within an observational prospective cohort. Healthy and mild preeclamptic pregnant women were studied at each trimester of gestation and three months postpartum. In addition, a group of non-pregnant women was studied into the follicular and luteal phases of the menstrual cycle. Furthermore, Ghrelin/LEAP-2 ratio was investigated in non-pregnant rats and at different periods of rat pregnancy. Human and rat serum ghrelin and LEAP-2 levels were determined using the commercially available ELISA kits. The Ghrelin/LEAP-2 ratio peak around the second trimester of gestation in healthy pregnant women (*p* < 0.05). Additionally, there were no statistically significant differences in Ghrelin/LEAP-2 ratio between healthy and preeclamptic pregnant women at each trimester of gestation (*p* > 0.05). The Ghrelin/LEAP-2 ratio in pregnant rat reached the peak around mid-gestation with a similar pattern to the human pregnancy. LEAP-2 was visualized by immunohistochemistry in human term placenta and rat placentas on days 12, 16 and 21 of pregnancy. In conclusion, this study provides the first evidence of a Ghrelin/LEAP-2 ratio peak around the half-way point of pregnancy onwards during human and rat pregnancy, and it might be associated with increased rates of weight gain during pregnancy. Thus, this study suggests that LEAP-2 and Ghrelin/LEAP-2 ratio might play an important role in maternal physiology adaptation of weight gain during pregnancy.

## 1. Introduction

Weight gain recommendations during pregnancy are essential to optimize fetal and maternal outcomes and to monitor long-term maternal and infant health conditions [1]. Additionally, independently of age, parity, race and based on pre-pregnancy body mass index (BMI) ranges, the Institute of Medicine (IOM) published the revised and appropriated gestational weight gain scale chart guidelines during pregnancy [2]. Thus, for normal pre-pregnancy BMI women at 40 weeks pregnant with one baby, the median weight gain recommendation is 14.5 kg (11.5–17.7) and weight gain varies in pre-pregnancy underweight and obese pregnant women [2]. Furthermore, research has shown that during pregnancy, body weight gain follows a non-linear shape, and pregnant women gain weight at a higher rate in the second compared with the first half of pregnancy [3].

On the other hand, normal pregnancy is considered as a “*diabetogenic state*” and different physiological adaptations take place, including energy homeostasis and metabolic changes that could be divided into the anabolic and catabolic phases [4]. In humans, the anabolic phase occurs during the first and second trimesters of pregnancy, and it is characterized by a marked increase of maternal fat accumulation and increased insulin sensitivity [5,6]. In contrast, the catabolic state occurs in the third trimester of human gestation, and it is characterized by increased adipose tissue lipolytic activity and insulin resistance [5,6]. Ghrelin, the endogenous natural ligand for the growth hormone secretagogue receptor 1α (GHS-R1α), also known as ghrelin receptor, is synthesized and secreted primarily by the gastric X/A cells and is an orexigenic peptide hormone mainly involved in the regulation of food intake and energy homeostasis [7]. In addition, ghrelin is involved in glucose and lipid metabolism and participates in the control of insulin release and insulin sensitivity [8,9,10]. During human pregnancy, serum ghrelin levels peak around mid-gestation and decrease significantly in the third trimester of pregnancy compared with the peak levels [11]. Additionally, different studies have shown a significantly negative correlation between circulating ghrelin levels and insulin resistance markers [10].

More recently, the liver-enriched antimicrobial peptide-2 (LEAP-2), primarily produced by the liver and small intestine, has been characterized as the endogenous GHS-R1α antagonist that blunts ghrelin action including food intake and body weight gain [12,13]. Moreover, previous reports have shown that circulating levels of LEAP-2 decreased during energy deficit to facilitate ghrelin actions, while, circulating LEAP-2 levels increase under postprandial conditions or food abundance to suppress ghrelin’s main functions involved in appetite and weight regulation [13,14].

Recently, Md Nurul Islam et al. demonstrated in a rat animal model that ghrelin and LEAP-2 cooperate to regulate feeding and energy homeostasis, and, similarly, results were obtained in observational research studies in humans [15,16]. Taking into consideration the findings from previous studies of LEAP-2 and ghrelin, rats have been extensively used as a model of pregnancy in similar studies to humans and could contribute to addressing specific physiological mechanisms because they share many biological features with humans. LEAP-2 levels and Ghrelin/LEAP-2 ratio have not been studied in normal and preeclamptic pregnancy and have not yet been studied in the animal model of rat pregnancy. Thus, the present study might contribute to elucidating the mechanisms related to the energy balance throughout pregnancy in humans and in the animal model of a rat.

## 2. Results

### 2.1. Results in Humans

Characteristics of the healthy and preeclamptic pregnant women and healthy non-pregnant women included in the present study are presented in Table 1, Supplementary Tables S1 and S2, respectively. During pregnancy, body mass index (BMI), insulin levels, HOMA index, total cholesterol, HDL, LDL, Very Low-Density Lipoprotein (VLDL) and triglycerides showed significant change in healthy pregnant women (*p* < 0.05) (Table 1). Similar changes were observed in BMI, total cholesterol, HDL, VLDL and triglycerides in preeclamptic pregnant women (*p* < 0.05) (Supplementary Table S1).

Differences of baseline characteristics between preeclamptic and healthy normotensive pregnant women were determined during the 1st trimester, 2nd trimester and 3rd trimester of pregnancy (Supplementary Table S3). Body mass index (BMI), blood glucose levels, HDL-c, VLDL, triglycerides, total cholesterol, systolic blood pressure (SBP), diastolic blood pressure (DBP) and medium blood pressure (MBP) at blood draw (mmHg), were significantly different between healthy and preeclamptic women in the third trimester of pregnancy (*p* < 0.05). Additionally, blood glucose, insulin levels and HOMA index, were significantly different between healthy and preeclamptic women in the 1st trimester and 2nd trimester of pregnancy (*p* < 0.05) (Supplementary Table S3).

**Figure 1:**
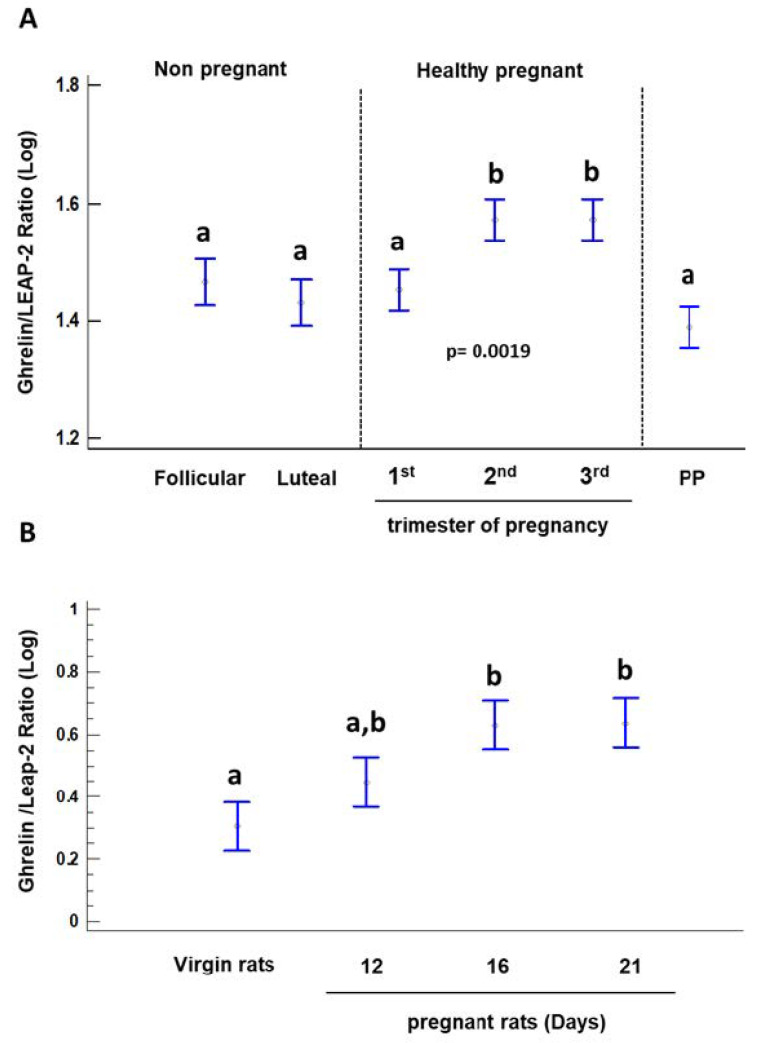
The Ghrelin/LEAP-2 ratio (Log) in healthy pregnant women at each trimester of gestation and during the follicular and luteal phases of the menstrual cycle (**A**) and Ghrelin/LEAP-2 ratio during pregnancy in rat (**B**). A *p* value of < 0.05 was considered as statistically significant. Mean Log [(Ghrelin ng/mL) × 10)]/Log [(LEAP-2 ng/mL) × 10] were used. Bars with no common letters are significantly different (*p* < 0.05).

On the other hand, serum ghrelin levels were significantly different during healthy pregnancy (*p* = 0.0000) (Table 1). Serum ghrelin levels decrease as pregnancy advances and reach its lowest levels at 3rd trimester of pregnancy as described elsewhere (*p* = 0.0000) (Table 1) [11]. Additionally, serum LEAP-2 levels showed the highest levels in the 1st trimester and decreases towards the end of gestation (*p* = 0.0000) (Table 1). The Ghrelin/LEAP-2 ratio reached its maximum at the 2nd trimester and persisted until the end of pregnancy in healthy pregnant women (*p* = 0.0019) (Figure 1, Table 1, and Supplementary Table S4). The Ghrelin/LEAP-2 ratio was not significantly different between the 1st trimester of healthy pregnancy, the follicular and luteal phase of the menstrual cycle and postpartum (*p* > 0.05) (Figure 1A and Supplementary Table S4).

In addition, serum Ghrelin and LEAP-2 levels and Ghrelin/LEAP-2 ratio were calculated in preeclamptic women (Supplementary Table S1). Serum ghrelin levels were significantly higher at the end of pregnancy in preeclamptic compared with healthy pregnant women, as described elsewhere (*p* = 0.0120) (Supplementary Table S3) [17,18,19]. Furthermore, serum LEAP-2 levels were significantly lower in the second (*p* = 0.0438) and third (*p* = 0.0015) trimesters of pregnancy in healthy compared with preeclamptic pregnant women (Supplementary Table S3). The Ghrelin/LEAP-2 ratio was not significantly different during each trimester of pregnancy between healthy and preeclamptic pregnant women (*p* > 0.05) (Supplementary Table S5).

Finally, Pearson’s correlation coefficient between Ghrelin/LEAP-2 ratio and the study biochemistry, clinical and anthropometric variables in healthy pregnant women during the 1st trimester, 2nd trimester and 3rd trimester of pregnancy and in healthy non-pregnant women during the follicular and luteal phases of the menstrual cycle were evaluated (Supplementary Tables S6 and S7, respectively). After log transformation of continuous variables, there were no significant correlations between Ghrelin/LEAP-2 ratio and each variable described in Supplementary Tables S6 and S7.

### 2.2. Results in Rat

Serum levels of ghrelin, LEAP-2 and Ghrelin/LEAP-2 ratio in virgin and pregnant rats are shown in Table 2 and Figure 1B.

### 2.3. Ghrelin and LEAP-2 Immunochemistry

#### 2.3.1. Human Ghrelin and LEAP-2 Immunostaining

**Figure 2:**
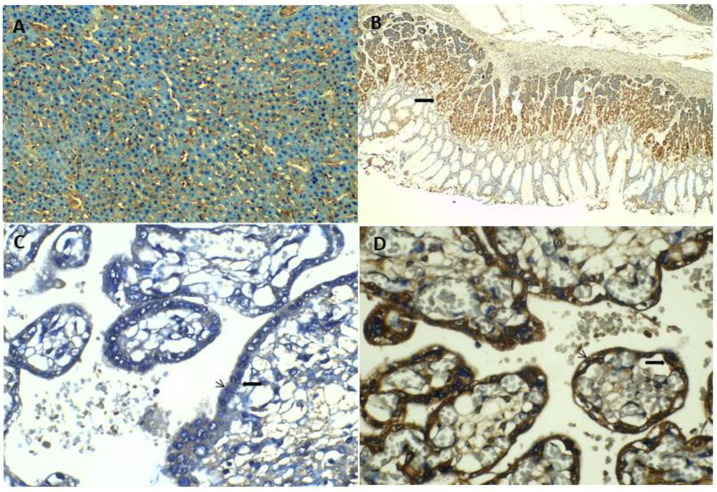
Immunolocalization of LEAP-2 and Ghrelin protein in human tissues. (**A**). LEAP-2 protein is immunolocated in liver tissue (10×). (**B**). Ghrelin protein is immunolocated in human gastric mucosa (filled arrow in (**B**) (4×). (**C**,**D**) show immunostaining of LEAP-2 (**C**) and Ghrelin (**D**), respectively in cytotrophoblasts (filled arrow) (40×) and syncytiotrophoblasts (arrows) (40×) sections of human term placentas.

Human LEAP-2 was visualized by immunohistochemistry in liver tissue and cytotrophoblast and syncytiotrophoblast of the human term placenta (Figure 2A,C). Additionally, immunostaining for human ghrelin was detected in gastric glandular cells, cytotrophoblast and syncytiotrophoblast of the human term placenta (Figure 2B,D).

#### 2.3.2. Rat Ghrelin and LEAP-2 Immunostaining

Rat LEAP-2 was visualized by immunohistochemistry in liver tissue and rat placentas on days 12, 16 and 21 of pregnancy (Supplementary Figure S1). Additionally, immunostaining for rat ghrelin was detected in gastric mucosa and rat placenta on days 12, 16 and 21 of pregnancy (Supplementary Figure S1). Positive staining for LEAP-2 and ghrelin were mainly found in the trophospongium in the zone of giant cells and trophospongium in rat placenta at day 12 of pregnancy. Immunohistochemical detection of LEA-2 at day 16 of rat pregnancy was determined in giant-cell layer and trophospongium of the junctional zone. Expression of ghrelin was predominantly present in the placental labyrinth zone at day 16 of rat pregnancy. In the rat placenta at day 22 of gestation, LEAP-2 and ghrelin were mainly found in the basal zone and labyrinth zone.

## 3. Discussion

The present study describes for the first time the serum profile of the LEAP-2 and the Ghrelin/LEAP-2 ratio in humans and rats throughout pregnancy. The results showed that humans and rats display a similar serum LEAP-2 profile during pregnancy, which consists of a significant decrease in serum LEAP-2 levels towards the end of pregnancy. Additionally, the serum ghrelin profiles during human and rat pregnancy were similar, as previously described [11,19,20,21,22,23]. Furthermore, the human Ghrelin/LEAP-2 ratio peaked around mid-gestation and was maintained until the end of pregnancy, and a similar pattern of Ghrelin/LEAP-2 ratio was observed in rat pregnancy. It is important to note that serum LEAP-2 levels were significantly lower in the second and third trimesters of pregnancy in healthy compare with preeclamptic pregnant women, but the Ghrelin/LEAP-2 ratios were not significantly different during each trimester of pregnancy between the two groups of pregnant women. Immunostaining of LEAP-2 was detected in human and rat placentas.

Previous studies have shown that maternally derived ghrelin levels change throughout pregnancy in humans and rats, and these findings suggest that ghrelin may play an important role in the maternal metabolic adaptations during pregnancy in order to ensure energy and substrate requirements for fetal growth during the intrauterine development and lactation [11,19,20,21,22,23]. Additionally, ghrelin has been shown to produce a wide variety of physiological effects, in particular the ability to promote food intake and weight gain [24,25,26]. According to the findings of the present study, previous research has shown that human serum ghrelin levels rose to their peak around mid-gestation, associated with elevated maternal weight gain during this period, and then ghrelin levels decrease with pregnancy progression, characterized by increased insulin resistance [11].

On the other hand, LEAP-2 was described recently as a GHS-R1α ligand, their serum concentration increased significantly upon refeeding, meanwhile, circulating ghrelin levels decreased under this metabolic condition [12,13,14]. Additionally, recent studies in vivo have demonstrated that LEAP-2 can inhibit the main functions of ghrelin, including the energy balance and body weight homeostasis [13]. In the current study, we showed that LEAP-2 was detected by immunohistochemistry in human and rat placentas, and these data suggest that LEAP-2 might be involved in placental regulation of ghrelin-mediated energy homeostasis during pregnancy; nevertheless, further studies must be carried out to demonstrate this possible relationship.

On the other hand, the results of this study showed that BMI increased significantly through each trimester of pregnancy, while maternal LEAP-2 serum levels, on the contrary, were significantly decreased in the mid-pregnancy until the end of human pregnancy. In addition, we found that the Ghrelin/LEAP-2 ratio is significantly increased around mid-pregnancy in human and rat pregnancies. Thus, the decrease in maternal LEAP-2 production through pregnancy, might favor different metabolic effects of ghrelin via activation of the GHS-R1α. Normal pregnancy can be considered as a *diabetogenic* state, manifested by insulin resistance in late pregnancy, with significant increases per trimester in maternal mean BMI and hypertriglyceridemia [4]. A recent study shows that LEAP-2 levels decrease significantly, and the Ghrelin/LEAP-2 ratio increases significantly in children with obesity, suggesting that this condition could favor the orexigenic, diabetogenic and lipogenic effects of ghrelin, similar to the physiological conditions that occur during pregnancy, as reported in the present study [27].

The results of this study showed that Ghrelin/LEAP-2 ratio did not differ between healthy and preeclamptic pregnant women at each trimester of pregnancy, while serum LEAP-2 levels in preeclamptic women compared to healthy pregnant women were significantly higher. Thus, despite the increase in ghrelin levels in preeclamptic women, ghrelin tone might be controlled through the elevation in LEAP-2 levels at the end of pregnancy.

This research paper is the first study of Ghrelin/LEAP-2 ratio in humans and rats during different phases of pregnancy and postpartum. The strength of the present study is that it is a nested case-control design within a prospective inception cohort in which 465 pregnant women, who were recruited between 11 and 13 weeks of gestation, were followed. The pregnant women were followed up in the three trimesters of pregnancy and at 3 months postpartum. Additionally, non-pregnant women with ovulatory menstrual cycles were studied in both phases of the menstrual cycle. The healthy pregnant women included in the study were randomly selected from the initial cohort. All the women, who developed mild preeclampsia, approximately 6.0%, were included in the study; however, no patient met the criteria for severe preeclampsia or eclampsia. Therefore, it would be important to develop studies in women with severe preeclampsia and eclampsia in which the different biomarkers analyzed in this study are determined. On the other hand, it would also be important to develop studies in women with diabetes mellitus, obesity, and low gestational weight, whose results could guide physicians in decision-making and early interventions to reduce the risk of adverse maternal perinatal outcomes. Another strength of the study is the research in the rat animal model that contributes to knowing the behavior of Ghrelin and LEAP-2 in pregnancy, and the presence of these biomarkers in the placenta. These results could be used as biomarkers for monitoring hypertensive disorders, to optimize the health care to prevent and treat women to reduce maternal and infant mortality. Furthermore, it is important to determine the Ghrelin/LEAP-2 ratio during pregnancy using the serum levels of Des-acyl Ghrelin (DAG) and acylated-Ghrelin (AG), respectively, to confirm the results of the present study. Additionally, the Ghrelin/LEAP-2 ratio could be studied in prevalent disorders that occur during pregnancy, such as obesity and diabetes.

Future studies on the Ghrelin/LEAP-2 ratio in pregnant women could contribute to the early prediction of adverse maternal and perinatal outcomes, and to the implementation of measures in early pregnancy to reduce the risk of adverse outcomes related to metabolic control of appetite and energy homeostasis, including miscarriage, gestational diabetes, preeclampsia, macrosomia, large for gestational age, stillbirth, congenital heart defects, spina bifida, preterm birth, cesarean delivery, postpartum hemorrhage and postpartum infection [28]. In addition, it is possible that indicators and risk prediction ranges of different adverse outcomes in pregnant women, can be implemented using this type of biomarkers.

In conclusion, this study has demonstrated for the first time that maternal serum levels of LEAP-2 change significantly throughout pregnancy in humans and rats. LEAP-2 levels decrease significantly towards the end of pregnancy, and this pattern might favor the orexigenic, diabetogenic and lipogenic effects of Ghrelin. Additionally, the Ghrelin/LEAP-2 ratio peaks around mid-gestation and is then maintained until the end of pregnancy, suggesting that this ratio may play an important role during pregnancy in the main functions of Ghrelin, including the energy balance and body weight homeostasis. Finally, the higher levels of LEAP-2 in preeclamptic pregnant women from mid-gestation as compared to normotensive pregnant women, might contribute to monitoring the metabolic tone of Ghrelin during pregnancy.

## 4. Methods and Materials

### 4.1. Ethics Approval and Consent to Participate

This study was performed according to the guidelines of the Helsinki Declaration, and the Ethical Committee of the Faculty of Medicine, Universidad Nacional de Colombia, approved all procedures of the current study (Reference: N°. 004-042/2021). Additionally, the written informed consent was obtained from all the participants before the study inclusion. We conducted this study at the university-affiliated Engativa Hospital, a tertiary care hospital in Bogota Colombia and the Departments of Physiology and Obstetrics and Gynecology of the School of Medicine, Universidad Nacional de Colombia.

### 4.2. Subjects

We conducted a case-control study nested within a prospective cohort study. Cases were women who developed mild preeclampsia during follow-up, and controls were age-matched normotensive pregnant women. Pregnant women were enrolled to this study at the first prenatal visit in the first trimester of pregnancy (11–13 weeks). Gestational age was calculated based on the last menstrual period and ultrasonography findings. A total of 25 healthy women, randomly selected from the original cohort study (n = 465) who delivered at term, normotensive with no medical and obstetrical complications during pregnancy, were studied during the first, second, and third trimesters of pregnancy and three months postpartum (Table 1). Additionally, pregnant women diagnosed with mild preeclampsia (n = 20) were included in the study (Supplementary Table S1). Approximately 6.0% of the women of the original cohort study who developed mild preeclampsia were included in the study; however, no patient met the criteria for severe preeclampsia or eclampsia.

All the women were followed throughout their pregnancies at each well visit to determine, biochemical, anthropometric, and clinical assessment. Also, age-matched healthy eumenorrheic women (n = 20) with a regular menstrual cycle (28–30 days) were studied during the follicular (cycle day 4 ± 1) and luteal (cycle day 22 ± 1) phases of the menstrual cycle.

Mild preeclampsia was diagnosed according to American College of Obstetricians and Gynecologists criteria: systolic blood pressure (SBP) of 140 mmHg or higher or diastolic blood pressure (DBP) of 90 mmHg or higher, measured at least 4 h apart with proteinuria detected by urine dipstick test and diagnosed at ≥34 weeks of gestation [29]. Women who, at the start of study, meet any of the following criteria were not eligible for the study: age <17 years old, multiple pregnancy, pre-gestational diabetes including type 1 and type 2 diabetes, use and abuse of drugs and alcohol, endocrine-related diseases, or other pre-existing medical conditions and major medical conditions.

### 4.3. Assays

#### 4.3.1. Sample Collection and Processing

Venous blood samples were obtained at each well visit from each woman between 7:30 a.m. and 8:30 a.m. after an overnight fast, and the serum was separated by centrifugation from the clot and stored at −80 °C until biochemical and hormonal analysis. Biochemistry determination of serum glucose, triglycerides, total cholesterol, High-Density Lipoprotein Cholesterol (HDL-c) and Low-Density Lipoprotein Cholesterol (LDL) levels were determined enzymatically in serum by using enzymatic methods (Spinreact-Girona, Spain). Serum progesterone concentrations were measured in eumenorrheic women using Roche Elecsys Progesterone Diagnostics Kit (Roche Elecsys 1010 Immunoanalyzer Boulder, Indianapolis, IN, USA). Additionally, basal insulin levels were measured using a chemiluminescence immuno assay (Roche Elecsys 1010 Immunoanalyzer Boulder, Denver, CO, USA). Homeostatic model assessment (HOMA) index was determined as described Matthews et al. in 1985 [30].

#### 4.3.2. Laboratory Animal Models

All procedures used in this experiment were approved by the Ethics Committee of the Universidad Nacional de Colombia in accordance with institutional guidelines for the care and use of experimental animals. Adult female Wistar rats with regular chow and water available ad lib were housed under controlled conditions of temperature, humidity, and illumination (12:12 light-dark cycle).

#### 4.3.3. Experimental Setting

Serum ghrelin and LEAP-2 levels were studied throughout pregnancy, according to methods described elsewhere [31]. Four groups of age matched pregnant rats were assigned to different experimental groups (n = 9 rats/group): three groups of rats fed ad libitum, were sacrificed on gestational days 12, 16 and 21 respectively. Additionally, a group of virgin rats fed ad libitum was studied. Animals were sacrificed following guidelines as previously mentioned, between 08:00 and 09:00, trunk blood was collected and centrifuged, and serum samples were stored at −80 °C until ghrelin and LEAP-2 quantification by ELISA.

#### 4.3.4. Human Ghrelin and LEAP-2 ELISA Assay

Human serum ghrelin levels were measured using a commercial ELISA KIT, according to the manufacturers’ instructions (Cat. No. MBS283919–MyBioSource, Southern California, San Diego, CA, USA). The lineal range of ghrelin ELISA assay was 0.156–10 ng/mL, while the intra-assay and inter-assay coefficients of variation were between <8% and <12%, respectively. Additionally, serum LEAP-2 levels were measured using a commercial Elisa KIT, according to the manufacturers’ instructions (Cat. No. MBS917663–MyBioSource, Southern California, San Diego, CA, USA). The lineal range of LEAP-2 ELISA assay was 0.312–20 ng/mL, while the intra-assay and inter-assay coefficients of variation were between <8% and <10%, respectively. Mean log [(Ghrelin ng/mL) × 10)]/Log [(LEAP-2 ng/mL) × 10] were calculated.

#### 4.3.5. Rat Ghrelin and LEAP-2 ELISA Assay

Rat ghrelin levels were measured using a commercial ELISA KIT, according to the manufacturers’ instructions (Cat. No. MBS2602044–MyBioSource, Southern California, San Diego, CA, USA). The lineal range of rat ghrelin ELISA assay was 0.156–10 ng/mL, while the intra-assay and inter-assay coefficients of variation were between <8% and <12%, respectively. Rat serum LEAP-2 levels were measured using a commercial ELISA KIT, according to the manufacturers’ instructions (Cat. No. MBS7229156–MyBioSource-Southern California, San Diego, CA, USA). The lineal range of rat LEAP-2 ELISA assay was 0.5–10 ng/mL, while the intra-assay and inter-assay coefficients of variation were between <8% and <10%, respectively. Mean log [(Ghrelin ng/mL) × 10)]/Log [(LEAP-2 ng/mL) × 10] were calculated.

#### 4.3.6. Ghrelin and LEAP-2 Immunochemistry

Human immunostaining for ghrelin and LEAP-2 were performed on human term placental tissue embedded in paraffin sections, obtained from the Pathology Department Services of the Faculty of Medicine at the Universidad Nacional de Colombia. Additionally, immunostaining for rat ghrelin and LEAP-2 were analyzed in paraffin sections of placentas on days 12, 16 and 21 of pregnancy.

Rabbit polyclonal antibody to human and rat ghrelin was utilized for immunohistochemistry (Catalog # PA1-1070-Invitrogen™-Thermo Fisher Scientific-Waltham, MA, USA, 02451). The positive control used for ghrelin immunohistochemistry was normal gastric mucosa tissue in humans and rats. Additionally, rabbit polyclonal antibody to human and rat LEAP-2 (Catalog # MBS2001931–MyBiosource-Southern California, San Diego, CA, USA) was utilized for immunohistochemistry. The positive control used for LEAP-2 immunohistochemistry was normal liver tissue in humans and rats.

For negative control, the primary antibody was omitted, followed by incubation with secondary antibodies and detection reagents. Immunohistochemistry analyses were performed with the UltraVision Quanto Detection System HRP DAB kit (Thermo-Fisher Scientific, Waltham, MA USA, 02451). Immunohistochemistry analyses were undertaken according to standard protocols with the UltraVision Quanto Detection System HRP DAB kit (Thermo-Fisher Scientific, Waltham, MA USA, 02451).

#### 4.3.7. Statistical Analysis

All statistical analysis was performed using R Statistical Software (version 3.4.0-Vienna, Austria). Continuous variables with normal distribution were presented as mean ± standard deviation of the mean (SD) while non-normal variables were reported as median (interquartile range [IQR]). Normal distribution of variables was verified using the Kolmogorov-Smirnov test. Variables with normal distribution were compared by Student’s *t* test and One-Way ANOVA was used when more than two groups were compared. Data were log transformed prior to the statistical analysis. To evaluate the Ghrelin/LEAP-2 ratio, mean Log [(Ghrelin ng/mL) × 10)]/ Log [(LEAP-2 ng/mL) × 10] was determined. The Pearson correlation coefficient was calculated to assess the correlation between normally distributed variables. Significant differences were considered when *p* < 0.05.

## Figures and Tables

**Table 1:** Characteristic of healthy normotensive pregnant women during the 1st trimester, 2nd trimester and 3rd trimester of pregnancy (n = 25).

**Variables**	**1st Trimester**	**2nd Trimester**	**3rd Trimester**	***p* Value** **(One-Way** **ANOVA Test)**
Maternal Age (years)	26.12 ± 6.94 (17.0–39.0)	_	_	
Gestational age (weeks)	12.62 ± 0.76 (11.1–14.0)	24.54 ± 0.68 (24.0–26.3)	34.61 ± 0.66 (34.0–36.2)	
BMI (kg/m^2^)	23.2042 ± 2.9601 (18.70–27.6)	25.428 ± 3.2033 (20.50–32.2)	27.388 ± 3.1351 (22.30–34.00)	0.0001
SBP (mmHg)	96.72 ± 10.6283 (80.00–120.00)	98.12 ± 9.7652 (80.00–110.00)	98.44 ± 9.1291 (80.00–116.00)	0.8069
DBP (mmHg)	63.36 ± 10.01 (50.00–92.00)	61.68 ± 5.74 (50.00–80.00)	61.24 ± 7.80 (50.00–82.00)	0.6181
MBP (mmHg)	74.48 ± 9.37 (60.00–98.00)	73.83 ± 6.01 (60.00–90.00)	73.64 ± 7.39 (62.67–93.33)	0.9216
Blood glucose (mg/dL)	74.24 ± 6.43 (63.00–91.00)	70.38 ± 5.63 (60.00–81.00)	71.72 ± 8.38 (59.00–93.00)	0.0914
Insulin (µUI/mL)	9.18 ± 4.60 (2.00–20.70)	10.98 ± 3.99 (5.30–18.40)	15.50 ± 7.97 (5.10–39.30)	0.0008
HOMA Index	1.70 ± 0.91 (0.346–4.19)	1.90 ± 0.73 (0.90–3.41)	2.56 ± 1.13 (0.78–4.89)	0.0025
Total cholesterol (mg/dL)	170.48 ± 33.21 (117.00–259.00)	221.84 ± 45.74 (150.00–342.00)	243.08 ± 53.64 (178.00–371.00)	0.0000
HDL (mg/dL)	56.88 ± 8.93 (37.00–78.00)	74.04 ± 10.67 (52.00–97.00)	70.56 ± 10.31 (49.00–86.00)	0.0000
LDL (mg/dL)	91.56 ± 32.68 (50.00–167.00)	107.04 ± 40.88 (41.00–201.00)	122.60 ± 49.98 (49.00–218.00)	0.0418
VLDL (mg/dL)	26.48 ± 11.48 (12.60–60.00)	40.26 ± 12.70 (21.00–64.00)	50.14 ± 15.12 (27.80–83.00)	0.0000
Triglycerides (mg/dL)	132.12 ± 56.72 (63.00–300.00)	202.32 ± 64.27 (106.00–324.00)	253.68 ± 78.66 (139.00–419.00)	0.0000
C–Reactive protein	6.632 ± 4.450 (0.20–15.40)	6.328 ± 4.831 (0.40–17.50)	6.452 ± 3.627 (1.00–13.2)	0.9694
Ghrelin (ng/mL)	4.5487 ± 0.8288 (2.913–6.171)	4.510 ± 0.712 (3.463–6.491)	3.6241 ± 0.733 (2.095–4.771)	0.0000
LEAP-2 (ng/mL)	1.3841 ± 0.1621 (1.162–1.707)	1.1363 ± 0.1688 (0.863–1.602)	0.9939 ± 0.1557 (0.718–1.261)	0.0000
^¥^ Ghrelin/LEAP-2 ratio	1.4867 ± 0.1365 (1.188–1.781)	1.6574 ± 0.2185 (1.32–2.224)	1.5932 ± 0.1865 (1.369–2.044)	0.0019

Abbreviations: BMI. Body mass index; HDL-C. High-Density Lipoprotein Cholesterol; VLDL, Very Low-Density Lipoprotein; SBP, Systolic blood pressure; DBP, Diastolic blood pressure; MBP, Medium blood pressure; LEAP-2, Liver-expressed antimicrobial peptide 2. A *p* value of < 0.05 was considered as statistically significant. ^¥^ Mean Log [(Ghrelin ng/mL) × 10)]/ Log [(LEAP-2 ng/mL) × 10] were used. One-Way ANOVA test was used for comparisons of continuous Log transformed values.

**Table 2:** Serum levels of ghrelin (ng/mL), LEAP-2 (ng/mL) and ^¥^ Ghrelin/LEAP-2 ratio in virgin and pregnant rats (Days 12, 16 and 21 of pregnancy).

Variables	Virgin (n = 9)	Day 12 (n = 9)	Day 16 (n = 9)	Day 21 (n = 9)	*p* Value (One-Way ANOVA Test)
Ghrelin (ng/mL)	0.4367 ± 0.1452 (0.208–0.632)	0.7233 ± 0.4790 (0.266–1.452)	1.1201 ± 0.2641 (0.729–1.49)	1.2561 ± 0.1907 (0.893–1.519)	0.0000
LEAP-2 (ng/mL)	14.1913 ± 7.4776 (4.651–28.766)	11.8578 ± 9.3876 (1.837–24.494)	5.9930 ± 3.6257 (2.000–11.413)	6.4120 ± 3.6651 (2.614–12.933)	0.0274
^¥^ Ghrelin/LEAP-2 ratio	0.3039 ± 0.1040 (0.138–0.437)	0.4472 ± 0.2608 (0.179–0.840)	0.6300 ± 0.1357 (0.419–0.873)	0.6376 ± 0.0990 (0.450–0.771)	0.0002

One-Way ANOVA test was used for comparisons of continuous values. Abbreviations: LEAP-2, Liver-expressed antimicrobial peptide 2. A *p* value of < 0.05 was considered as statistically significant. ^¥^ Mean Log [(Ghrelin ng/mL) × 10)]/ log [(LEAP-2 ng/mL) × 10] were used.

## Supplementary Materials

The following supporting information can be downloaded at: www.mdpi.com/article/10.3390/ijms23179514/s1.
