# Supplementary material for: Study of the Ghrelin/LEAP-2 Ratio in Humans and Rats during Different Phases of Pregnancy
Source: Int J Mol Sci. 2022 Aug 23;23(17):9514. doi: 10.3390/ijms23179514 (PMC9455743; doi:10.3390/ijms23179514)
Supplement: Supplementary file 1 [file ijms-23-09514-s001.zip › ijms-1862536-supplementary.pdf]

Article supplementary material

Study of Ghrelin/LEAP-2 ratio in human and rat during different phases of pregnancy

Maria Fernanda Garcés[1], Julieth Daniela Buell – Acosta[1], Edith Ángel – Müller[2], Arturo José Parada – Baños[2], Jaidy Acosta – Alvarez[3], Harold Felipe Saavedra – López[4], Roberto Franco – Vega[4], Luis Miguel Maldonado – Acosta[4], Franklin Escobar – Cordoba[5,6], Keydy Vásquez – Romero[7], Ezequiel Lacunza[8], Sofía Alexandra Caminos – Cepeda[9], Rubén Nogueiras[10,11], Carlos Dieguez[10,11], Ariel Iván Ruiz–Parra[2] and Jorge Eduardo Caminos[1]

[1]Department of Physiology, [2]Department of Gynecology and Obstetrics, [3]Department of Morphology, [4]Endocrinology Unit - Department of Internal Medicine,[5] Psychiatry Department, School of Medicine Universidad Nacional de Colombia, Bogotá 11001, Colombia. [6]Fundación Sueño Vigilia Colombiana, Bogotá 111211, Colombia. [7]Centro de Biomodelos CeBio – Departamento de Ciencias Biológicas, Universidad de los Andes – Colombia, Bogotá 111711, Colombia. [8]Centro de Investigaciones Inmunológicas Básicas y Aplicadas (CINIBA), Facultad de Ciencias Médicas, Universidad Nacional de La Plata, La Plata 1900, Argentina. [9]School of Medicine, Universidad Pompeu Fabra; Barcelona 08002, Spain. [10]CIBER Fisiopatología de la Obesidad y Nutrición, Instituto de Salud Carlos III, Madrid 28029, Spain. [11]Department of Physiology (CIMUS), School of Medicine - Instituto de Investigaciones Sanitarias (IDIS), Universidad de Santiago de Compostela 15782, Santiago de Compostela, Spain

Correspondence: Jorge Eduardo Caminos, MSc. PhD., Department of Physiology, School of Medicine, Universidad Nacional de Colombia, Carrera 30 No. 45-03,

Edificio 471 Oficina 406, Bogotá 11001, Colombia, E-mail: jecaminosp@unal.edu.co

Running title: The Ghrelin/LEAP-2 ratio during human and rat pregnancy

Key words: Ghrelin/LEAP-2 ratio, Pregnancy, endogenous GHSR antagonist.

Supplementary Table S1. Characteristics of preeclamptic women during the 1st trimester, 2nd trimester and 3rd trimester of pregnancy (n=20).

Variables	1st trimester	2nd trimester	3rd trimester	p value (One-Way ANOVA test)
Age (years)	23.6 ±5.3 (17.0 - 34.0)	-	-	
Gestational age (weeks)	12.3 ±0.64 (11.2 – 13.4)	24.4 ±0.55 (24.0 – 26.0)	34.9 ±0.87 (34.0 – 37.0)	
BMI (kg/m^2)	24.37 ±2.88 (20.86 – 31.24)	26.62 ±2.92 (22.38 – 32.60)	29.51 ±2.84 (24.30 – 34.48)	0.0000
SBP (mmHg)	101.91 ±8.54 (90.00- 120.00)	103.15 ±8.72 (90.00- 126.00)	107.68 ±12.51 (90.00- 145.00)	0.1411
DBP (mmHg)	65.09±7.37 (50.00- 80.00)	64.95±7.40 (56.00- 82.00)	66.05±7.44 (58.00- 80.00)	0.8680
MBP (mmHg)	77.36±7.37 (63.33- 93.33)	77.65±7.10 (67.33- 91.33)	79.91±8.19 (68.70- 101.70)	0.4779
Blood glucose (mg/dL)	80.80±6.38 (72.00 – 99.00)	76.65±7.60 (65.00 – 87.00)	73.53±6.57 (69.00 – 98.00)	0.0209
Insulin (µUI/mL)	11.83±4.01 (4.50 – 24.50)	14.37±5.02 (5.70 – 24.40)	15.38±6.71 (6.80 – 35.90)	0.0981

HOMA Index	2.35 ±0.82 (0.86- 4.78)	2.69 ±0.95 (1.06- 4.52)	3.16 ±1.56 (1.33- 7.98)	0.0825
Total cholesterol (mg/dL)	169.44±32.87 (92.00- 233.40)	216.96±42.60 (156.00- 355.00)	183.25±34.52 (117.60- 252.00)	0.0003
HDL (mg/dL)	51.82±12.16 (31.71- 81.91)	64.33±14.41 (42.68- 98.08)	47.54±11.66 (24.58- 70.00)	0.0002
LDL (mg/dL)	116.43±40.47 (52.30 - 207.49)	144.95±58.66 (78.87- 329.50)	129.29±32.52 (83.41- 200.50)	0.1240
VLDL (mg/dL)	23.66±7.66 (13.14 - 43.20)	35.20±10.45 (10.74 - 63.62)	27.06±16.35 (11.74 - 63.26)	0.0084
Triglycerides (mg/dL)	118.61±38.93 (65.70– 216.00)	176.28±52.06 (106.70– 311.00)	161.90±79.97 (54.60– 316.30)	0.0050
C–Reactive protein	6.41 ±3.71 (0.87 - 13.74)	7.71 ±2.90 (1.96 - 11.20)	5.50 ±3.88 (1.70 - 16.24)	0.1294
Ghrelin (ng/mL)	4.5242±0.6445 (3.1892– 6.1447)	4.5565±0.6170 (3.1976–5.9026)	4.3676±0.9837 (2.1289– 6.8210)	0.5290
LEAP-2 (ng/mL)	1.3779±0.2435 (0.8534- 1.999)	1.2588±0.2348 (0.972- 1.877)	1.204±0.2336 (0.7206- 1.6309)	0.0025
¥Ghrelin/LEAP-2 ratio	1.4697±0.1220 (1.276-1.779)	1.5154±0.1164 (1.231-1.672)	1.5126±0.1362 (1.237-1.788)	0.4573

One-Way ANOVA test was used for comparisons of continuous Log transformed values. Abbreviations: BMI. Body mass index; HDL-C. High-Density Lipoprotein Cholesterol; VLDL. Very Low-Density Lipoprotein; SBP. Systolic blood pressure (mmHg); DBP. Diastolic blood pressure (mmHg); MBP. Medium blood pressure (mmHg). LEAP-2, Liver-expressed antimicrobial peptide 2. A p value of < 0.05 was considered as statistically significant. ¥Mean log [(Ghrelin ng/mL) *10)]/ log [(LEAP-2 ng/mL) *10] were used.

Supplementary Table S2: Characteristics of non-pregnant women (n=20)

Variables	Healthy non-pregnant women	p value*
Age (years)	20.8 ±2.9 (18.0 - 29.0)	
BMI (kg/m²)	21.96 ±2.45 (18.37 – 25.54)	
SBP (mmHg)	110.222 ±8.3070 (92.00-125.00)	
DBP (mmHg)	72.6111±6.3722 (62.00-86.00)	
MBP (mmHg)	85.9196±4.2836 (79.00-95.70)	
Blood glucose (mg/dL)	86.95±6.3151 (77.0 - 97.0)	
Insulin (μUI/mL)	8.6316±4.6139 (2.10 - 17.20)	
HOMA Index	1.8321 ±1.0138 (0.4978 - 3.822)	
Total cholesterol (mg/dL)	159.389±30.1296 (118.00-224.70)	
HDL-C (mg/dL)	53.1474±8.8236 (39.46-69.63)	
LDL (mg/dL)	114.562±31.8487 (63.42-182.40)	
VLDL (mg/dL)	15.53±4.5555 (8.32–25.46)	
Triglycerides (mg/dL)	80.2611±21.0048 (54.90–127.30)	
C–Reactive protein	1.91 ±0.9908 (0.35 - 4.82)	
Follicular Progesterone (nmol/L)	0.4792±0.2425 (0.171–1.09)	0.0000
Luteal Progesterone (nmol/L)	7.3048±5.2051 (2.135–20.12)	
Follicular Ghrelin (ng/mL)	5.1624 ±0.6485 (3.594-6.318)	0.7117
Luteal Ghrelin (ng/mL)	5.2417 ±0.6974 (3.431-6.526)	

Follicular LEAP-2 (ng/mL)	1.4895 ±0.2054 (1.2208 - 1.8963)	0.0906
Luteal LEAP-2 (ng/mL)	1.6007 ±0.1995 (1.209 - 1.952)	
Follicular Log [(Ghrelin/LEAP-2)*10] ratio	1.467±0.087 (1.311 -1.581)	0.2396
Luteal Log [(Ghrelin/LEAP-2)*10] ratio	1.432±0.0860 (1.268 -1.585)	

Abbreviations: BMI, Body mass index; HDL-C, High-Density Lipoprotein Cholesterol; LDL, Low-Density Lipoprotein; VLDL, Very Low-Density Lipoprotein; SBP, Systolic blood pressure (mmHg); DBP, Diastolic blood pressure (mmHg); MBP, Medium blood pressure (mmHg); HOMA, Homeostatic model assessment; LEAP-2, Liver-expressed antimicrobial peptide 2. A p value of < 0.05 was considered as statistically significant. [¥]Mean Log [(Ghrelin ng/mL) *10)]/ Log [(LEAP-2 ng/mL) *10] were used.

Supplementary Table S3: Comparison of baseline characteristics between preeclamptic women and healthy normotensive pregnant women during the 1st trimester, 2nd trimester and 3rd trimester of pregnancy.

Variables	1st trimester	2nd trimester	3rd trimester
	Healthy vs Preeclamptic p value*	Healthy vs Preeclamptic p value*	Healthy vs Preeclamptic p value*
BMI (kg/m^2)	0.2883	0.1725	0.0175
SBP (mmHg)	0.0625	0.0717	0.0075
DBP (mmHg)	0.4167	0.0933	0.0255
MBP (mmHg)	0.2103	0.0518	0.0073
Blood glucose (mg/dL)	0.0007	0.0014	0.0001
Insulin (µUI/mL)	0.0113	0.0182	0.8427
HOMA Index	0.0049	0.0027	0.2647

Total cholesterol (mg/dL)	0.8846	0.7237	0.0000
HDL-C (mg/dL)	0.0710	0.0070	0.0000
LDL (mg/dL)	0.0259	0.0079	0.2928
VLDL (mg/dL)	0.2704	0.0833	0.0000
Triglycerides (mg/dL)	0.2625	0.0802	0.0000
C–Reactive protein	0.7487	0.0514	0.4507
Ghrelin (ng/mL)	0.9629	0.7771	0.0120
LEAP-2 (ng/mL)	0.7595	0.0438	0.0015
¥Ghrelin/LEAP-2 ratio	0.7133	0.1119	0.3498

The Mann–Whitney U test was used for comparisons of continuous Log transformed values. Abbreviations: BMI, Body mass index; HDL-C, High-Density Lipoprotein Cholesterol; LDL, Low-Density Lipoprotein; VLDL, Very Low-Density Lipoprotein; SBP, Systolic blood pressure (mmHg); DBP, Diastolic blood pressure (mmHg); MBP, Medium blood pressure (mmHg); HOMA, Homeostatic model assessment; LEAP-2, Liver-expressed antimicrobial peptide 2. A p value of < 0.05 was considered as statistically significant. ¥Mean Log [(Ghrelin ng/mL) *10)]/ Log [(LEAP-2 ng/mL) *10] were used.

Supplementary Table S4: Comparison of Ghrelin/LEAP-2 ratio during pregnancy and postpartum in healthy pregnant women and non - pregnant women.

Healthy Pregnant women

Healthy Pregnant

	Follicular	Luteal	1st trimester	2nd trimester	3rd trimester	Post-partum
Follicular		0.2376	0.6780	0.0008	0.0161	0.0471
Luteal	0.2376		0.4733	0.0000	0.0019	0.2640
1st trimester	0.6780	0.4733		0.0002	0.0049	0.0844
2nd trimester	0.0008	0.0000	0.0002		0.9828	0.0000
3rd Trimester	0.0161	0.0019	0.0049	0.9828		0.0002

Comparison of Ghrelin/LEAP-2 ratio during pregnancy and postpartum in healthy pregnant women and nonpregnant women. Statistical differences were evaluated through the Student t-test of independent samples assuming unequal variances. Log transformed values were used for testing differences variables. A p value of < 0.05 was considered as statistically significant. ¥Mean Log [(Ghrelin ng/mL) *10)]/ Log [(LEAP-2 ng/mL) *10] were used.

Supplementary Table S5: Comparison of Ghrelin/LEAP-2 ratio between healthy pregnant and preeclamptic women during each gestational period.

Preeclamptic women

Healthy Pregnant

	1st trimester	2nd trimester	3rd trimester
1st trimester	0.7119		
2nd trimester		0.1119	
3rd trimester			0.3498

Comparison of Ghrelin/LEAP-2 ratio between healthy pregnant and Preeclamptic women at each trimester of gestation. Statistical differences were evaluated through the Student t-test of independent samples assuming unequal variances. Log transformed values were used for testing differences variables. A p value of <0.05 was considered as statistically significant. ¥Mean Log [(Ghrelin ng/mL) *10)]/ Log [(LEAP-2 ng/mL) *10] were used.

Supplementary Table S6. Pearson's correlation coefficient between ¥Ghrelin/LEAP-2 ratio and study variables in healthy pregnant women during the 1st trimester, 2nd trimester and 3rd trimester of pregnancy.

Variable	Pregnant women 1st trimester		Pregnant women 2nd trimester		Pregnant women 3rd trimester	
	R-value	p-value	R-value	p-value	R-value	p-value
BMI (kg/m2)	-0.1084	0.6059	-0.2972	0.1491	-0.0130	0.9508
SBP (mmHg)	-0.0747	0.7228	-0.1147	0.5850	-0.1340	0.5231
DBP (mmHg)	-0.0089	0.9673	-0.0674	0.7489	0.0622	0.7675
MBP (mmHg)	-0.0355	0.8671	-0.1051	0.6171	-0.0183	09308
Blood glucose (mg/dL)	-0.1823	0.3038	-0.2496	0.2290	0.0795	0.7056
Insulin (µUI/mL)	-0.3104	0.1310	0.1618	0.4398	-0.1936	0.3538
HOMA Index	-0.3235	0.1147	0.0980	0.6411	-0.1593	0.4468
Total cholesterol (mg/dL)	0.1609	0.4422	-0.0681	0.7463	-0.1036	0.6222
HDL-C (mg/dL)	0.0366	0.8622	0.3607	0.0765	-0.3815	0.0599
LDL (mg/dL)	0.0724	0.7310	-0.1247	0.5527	0.0047	0.9823
VLDL (mg/dL)	-0.0210	0.9206	-0.1222	0.5607	-0.2825	0.1712
Triglycerides (mg/dL)	-0.0015	0.9942	-0.1242	0.5543	-0.2979	0.1480

Abbreviations: BMI, Body mass index; HDL-C, High-Density Lipoprotein Cholesterol; LDL, Low-Density Lipoprotein; VLDL, Very Low-Density Lipoprotein; SBP, Systolic blood pressure (mmHg); DBP, Diastolic blood pressure (mmHg); MBP, Medium blood pressure (mmHg); HOMA, Homeostatic model assessment; LEAP-2, Liver-expressed antimicrobial peptide 2. A p value of < 0.05 was considered as statistically significant. ¥Mean Log [(Ghrelin ng/mL) *10)]/ Log [(LEAP-2 ng/mL) *10] were used.

Supplementary Table S7. Pearson's correlation coefficient between Ghrelin/LEAP-2 ratio and study variables in healthy eumenorrheic women during the early follicular and mid - luteal phase of the menstrual cycle.

Variable	Follicular Phase		Luteal phase	
	R - value	p-value	R - value	p-value
BMI (kg/m²)	0.4186	0.0838	0.2382	0.3413
SBP (mmHg)	0.1018	0.6877	-0.3273	0.1850
DBP (mmHg)	-0.0593	0.8153	0.2762	0.2673
MBP (mmHg)	0.0112	0.9647	0.0480	0.8500
Blood glucose (mg/dL)	0.3447	0.1484	-0.1815	0.4570
Insulin (µUI/mL)	0.0644	0.7933	-0.2635	0.2757
HOMA Index	0.1153	0.6384	-0.2891	0.2299
Total Cholesterol (mg/dL)	0.1200	0.6245	-0.4027	0.0874
HDL-C (mg/dL)	0.2462	0.3097	-0.3580	0.1323
LDL (mg/dL)	0.2746	0.2553	0.0865	0.7247
VLDL (mg/dL)	-0.2374	0.3277	-0.5290	0.0199
Triglycerides (mg/dL)	0.0074	0.9759	0.0276	0.9106

Abbreviations: BMI, Body mass index; HDL-C, High-Density Lipoprotein Cholesterol; LDL, Low-Density Lipoprotein; VLDL, Very Low-Density Lipoprotein; SBP, Systolic blood pressure (mmHg); DBP, Diastolic blood pressure (mmHg); MBP, Medium blood pressure (mmHg); HOMA, Homeostatic model assessment; LEAP-2, Liver-expressed antimicrobial peptide 2. A p value of < 0.05 was considered as statistically significant. ¥Mean Log [(Ghrelin ng/mL) *10)]/ Log [(LEAP-2 ng/mL) *10] were used.

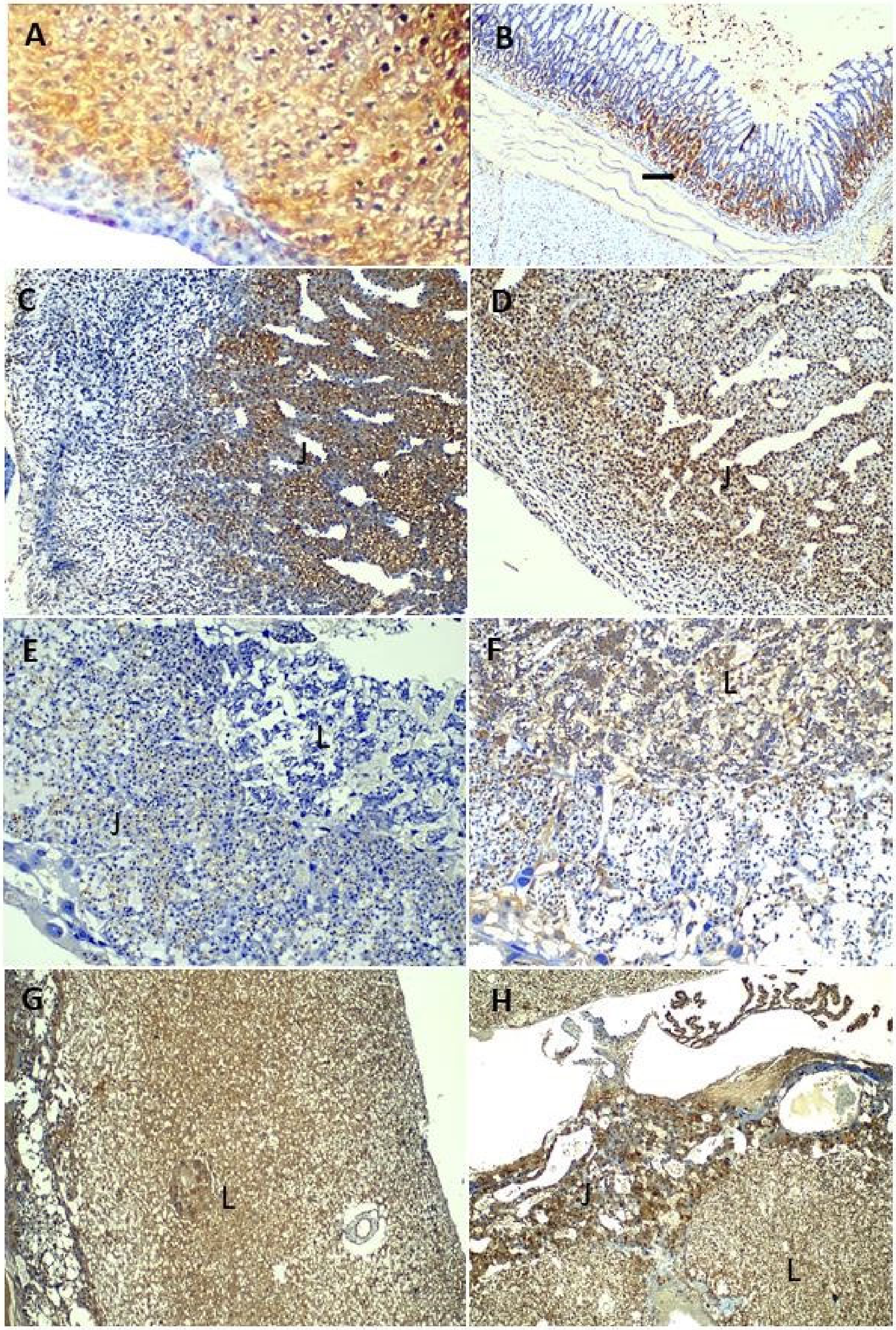

Supplementary Figure S1. Immunolocalization of LEAP-2 and ghrelin protein in rat tissues. Rat LEAP-2 was visualized by immunohistochemistry in liver tissue (A) and rat placentas on days 12 (C), 16 (E) and 21 (G) of pregnancy. Additionally, immunostaining for rat ghrelin was detected in gastric mucosa (B) (Filled arrows) and rat placenta on days 12 (D), 16 (F) and 21 (H) of pregnancy. Junctional zone (J) and labyrinth zone (L).
